# Using a blend of oilseed meals in the diets of Nile tilapia (*Oreochromis niloticus*): effects on the growth performance, feed utilization, intestinal health, growth, and metabolic-related genes

**DOI:** 10.1186/s12917-024-04373-5

**Published:** 2024-11-27

**Authors:** Ahmed A. Badran, Fawzy I. Magouz, Amr I. Zaineldin, Safaa E. Abdo, Asem A. Amer, Mahmoud S. Gewaily, Mahmoud A.O. Dawood

**Affiliations:** 1 Animal Production Department, Faculty of Agriculture, Kafrelsheikh University, Kafr El-Sheikh, 33516 Egypt; 2 Unit of Biochemistry, Nutritional Deficiency Diseases and Toxicology, Agriculture Research Center, Animal Health Research Institute (AHRI-DOKI), Kafr El-Sheikh branch, Giza, 85871 Egypt; 3 Genetics and Genetic Engineering, Department of Animal Wealth Development, Faculty of Veterinary Medicine, Kafrelsheikh University, Kafr El-Sheikh, 33516 Egypt; 4 Department of Fish Nutrition and Feed Technology, Central Laboratory for Aquaculture Research, Agricultural Research Center, Abbassa, Abo-Hammad, Sharqia, Giza, 85871 Egypt; 5 Department of Anatomy and Embryology, Faculty of Veterinary Medicine, Kafrelsheikh University, Kafr El-Sheikh, 33516 Egypt; 6 The Center for Applied Research on the Environment and Sustainability, The American University in Cairo, New Cairo, Cairo 11835 Egypt

**Keywords:** Aquafeed, Alternative ingredients, Digestion, Metabolism, Tilapia

## Abstract

In this study, Nile tilapia were fed a blend of oilseed meals (BOM) that includes cottonseed meal (CSM), linseed meal (LSM), sesame meal (SSM), and sunflower meal (SFM) at a ratio of 1 CSM: 1 LSM: 1 SSM: 1 SFM. Six diets were formulated where the first diet included FM and SBM as protein sources and considered the positive control diet (FM). Another five FM-free diets were formulated, where SBM was substituted with BOM and included at 0, 100, 200, 300, and 400 g/kg diet. After 90 days, the FBW, WG, and PER were markedly increased while FCR decreased by FM-based diet and BOM at 0, 100, or 200 g/kg compared to fish-fed BOM at 300, and 400 g/kg (*P* < 0.05). The groups treated with BOM at 100–200 g/kg demonstrated considerable impairments, followed by those treated with BOM at 300 g/kg. Furthermore, fish given BOM at 400 g/kg had significantly less intestinal histological characteristics than the other groups. The relative expression of the *IGF-1*, *GHR1*, *FABP*, and *CCK* genes were downregulated in tilapia-fed BOM at 200, 300, and 400 g/kg compared to fish-fed FM-based diet (*P* < 0.05). The relative cost of feed per kg fish gain showed 4.42, 7.11, 8.14, 10.32, and 8.10% reduction rates in fish-fed SBM, or BOM at 100, 200, 300, and 400 g/kg. In conclusion, dietary BOM can be incorporated in Nile tilapia diets at up to 200 g/kg without affecting growth performance or feed utilisation. High inclusion levels (300 and 400 g/kg) may impair growth performance and feed utilisation by disrupting intestinal histological characteristics and reducing expression of growth and metabolic genes (GHR1, IGF-1, FABP, and CCK) in the liver.

## Introduction

The aquaculture sector has expanded recently to meet the dramatic needs of humanity for healthy and safe seafood [1–3]. Alongside the aquafeed industry has developed to produce optimum nutritional formulas for healthy and productive aquatic animals [4]. The feed cost contributes 60–70% of the total production cost which limits the profitability and sustainability of aquaculture [5]. Traditionally, fish meal (FM) and soybean meal (SBM) have been used for formulating palatable and nutritious feeds [3, 6]. However, low availability, high prices, and huge demand reduced their usage in the aquafeed industry [7]. After FM, SBM is the most nutritious protein source ingredient associated with the rich amino acid profile, digestibility, and availability [8, 9]. Nevertheless, the high prices of SBM and the shortage of local cultivation challenged the aquafeed industry in some countries [10, 11].

Egypt is one of the active players in the aquaculture industry, especially Nile tilapia (*Oreochromis niloticus*) farming, and is ranked as the third largest producer of tilapia globally [12, 13]. Nile tilapia can grow quickly under captivity, meet the consumer’s demand, and resist semi-intensive and intensive systems [14, 15]. This huge production of tilapia requires sustainable protein sources and ingredients [16, 17]. Tilapias have omnivorous feeding habits that allow utilizing animal and plant protein sources [18]. Locally, alternative plant protein sources were suggested to replace FM and SBM in tilapia feeds [19–21]. The generated by-products of oilseed extraction are relatively low cost compared to FM and SBM with high nutritional value and were successfully included in aquatic animals’ diets [22–24]. Cottonseed meal (CSM) is a by-product of dehulled cottonseed after oil extraction and contains suitable protein content and amino acids [25, 26]. Dietary CSM was included in the diets of Nile tilapia to replace FM [27–29] or SBM [30] without affecting growth performance and productivity. Further, sesame seed meal (SSM) primarily results after sesame oil extraction, and the generated SSM cake can be utilized in fish feeds [31–33]. Y-X Guo, X-H Dong, B-P Tan, S-Y Chi, Q-H Yang, G Chen and L Zhang [34] and O Olude, F George and W Alegbeleye [35] reported that dietary SSM can be included in Nile tilapia diets without impairing growth performance and feed utilization. Sunflower meal (SFM) is another by-product resulting from oil extraction with a suitable protein and amino acid profile [36–38]. Dietary SFM was included in the diets of Nile tilapia without decreasing the growth performance as reported by EO Ogello, EM Kembenya, CM Githukia, CN Aera, JM Munguti and CS Nyamweya [39] and MS Hassaan, MA Soltan, EY Mohammady, MA Elashry, ER El-Haroun and SJ Davies [40]. Linseed meal (LSM) is another by-product that can be used as a protein source in feeds [41]. D Pianesso, FR Goulart, TJ Adorian, PI Mombach, JS de Lima, TS dos Santos and LP da Silva [42] reported that LSM can be included in the diets of silver catfish (*Rhamdia quelen*) as FM replacer up to 40% without affecting the growth performance and feed utilization. However, no investigations tested the possibility of including LSM in Nile tilapia diets. Most of the earlier efforts tested the possibility of including by-products of oil extraction meals in tilapia diets as FM replacers either individually or in composites [43]. In this regard, DMSD El-Saidy and MMA Gaber [44] reported that a plant protein mixture composed of SBM, CSM, SFM, and LSM could replace FM inclusion without affecting the performances of Nile tilapia. However, no studies were conducted to test the replacement of SBM with a blend of oilseed meals (CSM + SSM + FSM + LSM) in Nile tilapia diets. Hence, this study aimed at the evaluation of partial replacement of SBM with a blend of CSM + SSM + SFM + LSM on the growth performance, feed utilization, and intestinal health of Nile tilapia.

## Materials and methods

### Test diets

Six test diets were formulated to be isolipidic (5.32% total lipids) and isonitrogenous (30.21% crude protein) (Table 1). The first diet was formulated using fish meal (FM) and soybean meal (SBM) as protein sources and considered the positive control diet (FM). Another five FM-free diets were formulated where SBM was substituted with a blend of cottonseed meal (CSM), linseed meal (LSM), sesame meal (SSM), and sunflower meal (SFM) at 0, 100, 200, 300, and 400 g/kg diets. The mixture of oilseed meals was mixed at the rate of 1 CSM: 1 LSM: 1 SSM: 1 SFM. In the presence of yellow corn, gluten meal, wheat middling, wheat bran, monocalcium phosphate, vitamins and minerals, vitamin C, plant oil, fish oil, lysine, and methionine, all ingredients were well mixed. Betaine was added to the five FM-free diets to enhance the palatability of diets vs. FM-based diet [45]. Water was added to the contents of the six diets at 35–40% then the ingredients were homogeneously mixed again. Diets were pelleted with the laboratory pelletizing machine to produce 2–3 mL pellets. The obtained pellets were air-dried at room temperature and kept at 4 °C until used. The chemical composition including the total protein, lipids, ash, fibers, and amino acids were determined according to AOAC [46].

## Experimental procedures and final sampling

The trial was conducted at the Department of Fish Nutrition and Feed Technology, Central Laboratory for Aquaculture Research, Agricultural Research Center, Sakha Branch, Kafrelsheikh, Egypt. First, the receiving tank (1000 L), 18 glass aquaria (70 L), and husbandry tools were sterilized and washed with fresh water to keep a high level of biosecurity during the trial. Afterward, all male Nile tilapia juveniles were obtained from a commercial hatchery at the International Road to Baltim City, Kafrelsheikh, and then gently transported to the wet laboratory. Fish were acclimatized for two weeks where water was exchanged daily with free dechlorinated water and offered the FM-based diets twice daily at 3%. Then fish was stocked in 18 glass aquaria at 15 fish per aquarium at an initial weight of 10.97 ± 0.04 g/fish. The glass aquaria were supplied with continuous aeration and water was exchanged daily with dechlorinated fresh water. Fish offered the experimental test diets up to the satiation level twice daily (8 am and 3 pm) and consumed feed was recorded to calculate the feed utilization. The water quality indices were kept at 27.22 ± 0.21 °C for temperature; 7.42 ± 0.14 for pH; 5.43 ± 0.21 mg/L for dissolved oxygen; and 0.02 ± 0.001 mg/L for total ammonia nitrogen. The trial was continued under these conditions for 90 days then all fish were starved for 24 h before the final sampling. On sampling day, all fish were sedated (100 mg MS-222/L), then weighed individually and counted. Three fish per aquarium were randomly collected and bled from the caudal vein using 2.5 mL syringes. The collected blood was kept at 4 ºC for two hours then centrifuged at 3000 rpm for 15 min at 4 ºC (SCILOGEX, Model: DM0412, USA) for serum collection which was separated and stored at -20 ºC until used. After blood collection, the intestines were separated and kept in formalin (10%) for the histological study. The liver was also dissected and kept in liquid nitrogen until frozen at -80 ºC for gene expression. Another three fish per aquarium were collected and weighed then kept in the freezer at 20 ºC for the analysis of the body composition. Three fish per aquarium were collected and weighed, and their body length was recorded to calculate the condition factor (CF). Further, the liver and viscera were dissected and weighed to calculate the hepatosomatic index (HSI) and viscera somatic index (VSI).

CF = body weight (g) / (fish length)^3^ (cm)^3^ × 100.

HSI (%) = liver weight (g) / body weight (g) × 100.

VSI (%) = viscera weight (g) / body weight (g) × 100.

**Table 1:** Formulation and composition of the test diets

Ingredients	FM	Inclusion level (g/kg)								
		0	100		200		300		400	
Fish meal (61% CP)	80	0	0		0		0		0	
Soybean meal (44% CP)	340	420	370		290		230		150	
Cottonseed meal	0	0	25		50		75		100	
Linseed meal	0	0	25		50		75		100	
Sesame meal	0	0	25		50		75		100	
Sunflower meal	0	0	25		50		75		100	
Yellow corn	160	170	180		180		188		190	
Corn gluten	60	80	60		60		60		60	
Wheat middling	149.5	137.5	97.5		76.5		29.5		19.5	
Wheat bran	144	116	116		116		116		104	
Monocalcium phosphate	20	20	20		20		20		20	
Vitamins and minerals^1^	20	20	20		20		20		20	
Vitamin C	0.5	0.5	0.5		0.5		0.5		0.5	
Soyabean oil	18	20	20		18		20		20	
Fish oil	8	12	12		15		12		12	
Lysine	0	1	1		1		1		1	
Methionine	0	1	1		1		1		1	
Betaine	0	2	2		2		2		2	
Total	1000	1000	1000		1000		1000		1000	
Chemical composition										
Crude protein (%)	30.16	30.25	30.18		30.06		30.45		30.17	
Crude lipids (%)	5.15	5.29	5.25		5.16		5.21		5.31	
Ash (%)	6.84	6.65	6.41		6.63		6.73		6.81	
Fibers (%)	5.41	5.22	5.36		5.42		5.32		5.18	
NFE (%)^2^	52.44	52.60	52.80		52.73		52.29		52.53	
Gross energy (MJ/kg)^3^	18.17	18.27	18.28		18.20		18.24		18.25	
Protein/energy (P/E) ratio (g protein/MJ)^4^	16.60	16.55	16.51		16.51		16.70		16.53	
Amino acid (g/kg)										
Arginine	1.71	1.62	1.60		1.66		1.67		1.70	
Histidine	0.64	0.61	0.64		0.65		0.67		0.68	
Isoleucine	0.93	0.93	0.96		0.98		0.96		0.92	
Leucine	2.09	1.99	2.11		1.98		2.03		2.09	
Lysine	1.48	1.43	1.45		1.44		1.42		1.37	
Methionine	0.84	0.82	0.84		0.86		0.84		0.82	
Phenylalanine	1.19	1.17	1.28		1.29		1.23		1.22	
Threonine	1.22	1.19	1.21		1.16		1.18		1.15	
Valine	1.16	1.15	1.13		1.14		1.12		1.11	

FM: fish meal-based diet. All ingredients were obtained from Feed Control Co., Ltd. (Damro, Sidi Salem, Kafrelsheikh, Egypt) in dry powdered form.

^1^Vitamin mixture (vitamin C free) and mineral mixture (mg/kg premix) according to FF El-Desouky, MA Ibrahim, IM Abd El-Razek, E-SM El-Nabawy, AA Amer, AI Zaineldin, MS Gewaily and MAO Dawood [47].

^2^Nitrogen free extract (NFE) = 100 - (crude protein + crude lipids + fibers + ash).

^3^Gross energy was calculated based on protein, lipid, and carbohydrate values as 23.6, 39.5, and 17.2 KJ/g, respectively.

^4^Protein/energy (P/E) ratio = (crude protein / gross energy) × 10 [48].

## Diet and fish body composition

The composition of the test diets and fish body was determined by following AOAC [46]. For the ash, samples were burnt in the Muffle furnace (Thermolyne Corporation, Dubuque, Iowa, USA) at 550 °C for 6 h (method 930.30 [46]) while the moisture content was determined using oven-drying (GCA, model 18EM, Precision Scientific Group, Chicago, IL, USA) at 110 °C to reach the constant weight (method 952.08 [46]). Crude protein (N factor = 6.25) was analyzed via the Kjeldahl apparatus (Labconco, Labconco, Kansas, MO, USA) (method 992.23 [46]). Crude fat was extracted using the Soxhlet extraction method (Lab-Line Instruments, Melrose Park, IL, USA) (method 948.15 [46]). The system used for detecting the amine acids profile was high performance Amino Acid analyzer (Biochrom 30).

## Blood biochemistry

Serum total protein was measured using diagnostic reagent kits (Spectrum, Egyptian Company for Biotechnology, Egypt) at the wavelength of 546 nm according to D Cannon, O I and I JA [49]. Albumin was measured colorimetrically using diagnostic reagent kits (Biodiagnostic Co. Egypt) at the wavelength 630 nm, according to BT Doumas, DD Bayse, RJ Carter, T Peters and R Schaffer [50]. Globulin content was calculated mathematically. Aspartate aminotransferase (AST) and alanine aminotransferase (ALT) (Biodiagnostic Co. Egypt.) were determined colorimetrically at the wavelength 505 nm according to S Reitman and S Frankel [51]. Creatinine and urea contents were detected by the following H Bartles, M Bohmer and C Heirli [52] and J Fawcett and J Scott [53] using commercial kits (Biodiagnostic Co. Egypt.).

## Intestinal histology

After 24 h, the collected intestinal (anterior, middle, and posterior) samples were transferred from 10% neutral buffered formalin to 70% alcohol. The intestine samples were then dehydrated in ascending graded series of ethanol, cleared in xylene, and impregnated and embedded in paraffin wax [54]. Sections of 5 μm were cut using Leica rotatory microtome (RM 20352035; Leica Microsystems, Wetzlar, Germany) and mounted on glass slides. The prepared tissue sections were subjected to conventional staining of hematoxylin and eosin (H&E) according to MS Gewaily and MM Abumandour [55]. The stained sections were examined under a light microscope (Olympus, Tokyo, Japan). The stained sections were examined under a light microscope (Leica DM500; Leica Microsystems, Heerbrugg, Switzerland). The morphometric analysis utilized an automated image analysis system (ImageJ; Bethesda, MD, USA) to assess villus height and width, crypt depth, and muscularis thickness as outlined by CA Schneider, WS Rasband and KW Eliceiri [56]. Measurements were conducted in micrometers (µm). The obtained data were subjected to statistical analysis.

### Real-time PCR (qPCR)

The total RNA was extracted from liver samples (3 samples/ treatment) using easy-red (iNtRON Biotechnology, Inc.) according to the instructions included. The RNA quality was checked using ethidium bromide-stained agarose gel electrophoresis. RT reaction was done to synthesize the complementary cDNA from the extracted mRNA using Thermo Scientific first strand cDNA synthesis kit (TOPscript™ RT Dry MIX) following the kit procedures. The gene amplification was done in PikoRealTM 24, Thermo-scientific, Waltham, MA, USA, TCR0024). Specific primer pairs were used for the assessment of some feed intake-related gene (*CCK*), growth-related genes (*GHR1 and IGF-1*), and fat metabolism-related gene (*FABP*) in the liver tissue. The gene amplification was done in a Real-Time PCR System (PikoReal, Thermoscientific, TCR0024) and using the 2x -Lo-Rox- SYBR green kits (Applied Biotechnology, Egypt). The amplification condition and the reaction mix were done according to S El-Kassas, N Aljahdali, SE Abdo, FS Alaryani, EM Moustafa, R Mohamed, W Abosheashaa, E Abdulraouf, MA Helal, ME Shafi, et al. [57], and genes specific annealing temperature listed in Table 2. The real-time thermocycler conditions were 30 s of pre-denaturation at 95 °C, followed by 40 cycles of denaturation at 95 °C for 10 s and annealing at 60 °C for 30 s. The samples were prepared in a total reaction volume of 20 µl including 10 µl of SensiFast™ SYBR master mix (Bioline, United Kingdom), 2 µl of cDNA, 0.5 µM of each primer, and water up to 20 µl. Samples were run in duplicates. The mRNA relative expression was calculated as a fold change according to KJ Livak and TD Schmittgen [58], where the data were normalized against two housekeeping genes, beta-actin (*β-actin*) and elongation factor-1α (*ef-1α*), and the control group.

**Table 2:** The primer sequence used in real-time PCR

Gene	Primers	Accession no.	Amplification efficiency (%)	Annealing Tem/ °C	Ref.	
*Elongation factor-1α (ef-1α)*	F: TCAACGCTCAGGTCATCATC R: ACGGTCGATCTTCTCAACCA	XM_003458541	95.25261038	60	[59]	
*β-actin*	F: CAGCAAGCAGGAGTACGATGAG R: TGTGTGGTGTGTGGTTGTTTTG	XM_003455949.2	86.9558817	60	[60]	
*GHR1*	F: CAGACTTCTACGCTCAGGTC R: CTGGATTCTGAGTTGCTGTC	AY973232.1	93.90122652	61	[61]	
*IGF-1*	F: GTTTGTCTGTGGAGAGCGAGG R: GAAGCAGCACTCGTCCACG	Y10830.1	94.44544162	61	[61]	
*FABP3*	F: CAAGCCCACCACCATCATCT R: TTCCCGTCCTCTATCGTGACA	XM_003444047.5	96.13504153	60	[62]	
*Cholecystokinin*	F: CAGAAACTCCACGGCAAACA R: TCATACTCCTCTGCACTGCG	NM_001279730.1	95.96104163	60	[63]	

*GHR1*: growth hormone receptors 1. *IGF-1*: insulin-like growth factor 1. *FABP3*: fatty acid binding protein 3.

## Economic feasibility analysis

The economic analysis was performed according to M Abdel-Tawwab, RH Khalil, AA Metwally, MS Shakweer, MA Khallaf and HMR Abdel-Latif [64]. The prices of the local market for used ingredients were applied. The rate of USD to Egyptian pound (EGP) at the time of terminating the trial was 1 USD = 48 EGP. The following equations were used:

Cost reduction per ton gain (USD) = feed cost per kg gain of the positive control diet (FM) – feed cost per kg gain of SBM or BOM-based diets (USD).

Cost reduction per kg gain (%) = 100 (cost reduction per kg gain [USD] in SBM or BOM-based diets/feed cost per kg gain of the FM-based diet [USD].

## Equations for growth performance and statistical analysis

According to [65]: Weight gain (WG, %) = [(FBW – IBW) ×100] / IBW,

Specific growth rate (SGR; % g / day) = 100 × [(LnFBW - LnIBW) / 90 days],

Feed conversion ratio (FCR) = FI / WG,

Protein efficiency ratio (PER) = [WG (g) / PI (g)] × 100,

Survival (%) = [final number / initial number] × 100.

Where FBW: final weight (g); IBW: initial weight (g); WG: weight gain (g); Ln: natural log.; FI: feed intake (g/fish); PI: protein intake (g).

Shapiro-Wilk and Levene’s tests confirmed the normal distribution and homogeneity of variance. The obtained data was subjected to a one-way ANOVA to evaluate the effect of the proposed six diets on the designated parameters. Differences between the means were tested at the 5% probability level using a Duncan test as a post-hoc test. All the statistical analyses were conducted with SPSS version 22 (SPSS^®^ Inc., IL, USA). Excluding the FM diet, polynomial contrasts were used to detect the quadratic effects of various replacement levels of the blend of oilseed meals on WG (%) using a polynomial regression analysis [66].

## Results

### Growth performance

The effects of the dietary blend of oilseed meals (BOM) on the growth performance and feed efficiency of Nile tilapia are shown in Table 3. Out of the final weight (FBW), weight gain (WG), feed conversion ratio (FCR), and protein efficiency ratio (PER), the results showed no marked effects on the final weight, specific growth rate, and feed intake (*P* > 0.05). However, the FBW, WG, and PER were markedly increased by fish meal-based diet (FM) and BOM at 0, 100, or 200 g/kg compared to fish-fed BOM at 300, and 400 g/kg (*P* < 0.05). Further, the FCR showed a marked reduction by FM and BOM at 0, 100, or 200 g/kg compared to fish-fed BOM at 300, and 400 g/kg (*P* < 0.05). No effects of the test diets were seen on the survival rate which showed a high rate (97.78–100%) (*P* > 0.05).

**Fig. 1:**
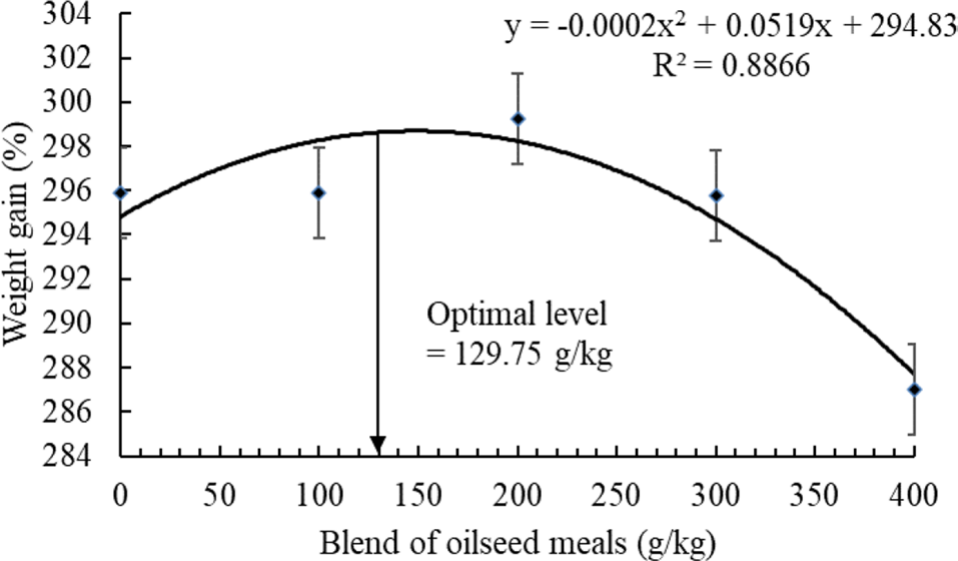
Polynomial regression analysis (*P* < 0.05) between weight gain of Nile tilapia after feeding with dietary blend of oilseed meals for 90 days. Values are described as means ± SE

The regression analysis indicated positive quadratic effects on the WG (R² = 0.8866) (Fig. 1). Accordingly, dietary BOM can be included up to 129.75 g/kg based on the results of WG.

**Table 3:** Effects of dietary blend of oilseed meals on the growth performance and feed efficiency of Nile tilapia

Items	FM	Inclusion level (g/kg)				
		0	100	200	300	400
IBW (g)	10.80 ± 0.20	11.05 ± 0.02	10.98 ± 0.02	11.00 ± 0.04	11.02 ± 0.02	10.98 ± 0.02
FBW (g)	43.33 ± 2.84^a^	43.72 ± 2.71^a^	43.83 ± 1.52^a^	43.54 ± 1.12^a^	42.65 ± 2.23^b^	42.29 ± 0.60^b^
WG (%)	301.00 ± 22.85^a^	295.87 ± 25.18^a^	299.26 ± 13.19^a^	295.76 ± 9.47^a^	287.01 ± 20.69^b^	285.22 ± 5.97^b^
SGR (%/day)	1.54 ± 0.06	1.53 ± 0.08	1.54 ± 0.03	1.53 ± 0.03	1.50 ± 0.06	1.50 ± 0.02
FI (g/fish/90 days)	44.67 ± 0.67	43.33 ± 0.67	44.00 ± 1.15	44.67 ± 0.88	44.67 ± 1.20	45.00 ± 0.58
FCR	1.39 ± 0.10^b^	1.33 ± 0.11^b^	1.35 ± 0.05^b^	1.37 ± 0.07^b^	1.43 ± 0.12^a^	1.44 ± 0.02^a^
PER	2.42 ± 0.17^a^	2.49 ± 0.16^a^	2.47 ± 0.11^a^	2.43 ± 0.13^a^	2.34 ± 0.22^b^	2.30 ± 0.03^b^
Survival (%)	100.00 ± 0.00	100.00 ± 0.00	100.00 ± 0.00	97.78 ± 2.22	100.00 ± 0.00	97.78 ± 2.22

Values are described as means ± SE. Different letters indicate significant differences (*P* < 0.05). FM: fish meal-based diet; IBW: initial body weight (g); FBW: final body weight (g); WG: weight gain (%); SGR: specific growth rate (%/day); FI: feed intake (g/fish); FCR: feed conversion ratio; PER: protein efficiency ratio.

### Body composition and somatic index

The effects of dietary BOM on the body composition and somatic index of Nile tilapia are shown in Table 4. No marked effects on the moisture and ash contents (*P* > 0.05). However, the crude protein content was markedly increased by FM and BOM at 0–100 g/kg compared to fish-fed BOM at 200, 300, and 400 g/kg (*P* < 0.05). The highest crude protein content was observed in fish-fed FM-based diet while the lowest was in fish-fed BOM at 200, 300, and 400 g/kg (*P* < 0.05). The ether extract showed a marked reduction by FM and BOM at 0–100 g/kg compared to fish-fed BOM at 200, 300, and 400 g/kg (*P* < 0.05). No marked effects on the somatic index (CF, HSI, and VSI) (*P* > 0.05).

**Table 4:** Effects of dietary blend of oilseed meals on the body composition and somatic index of Nile tilapia

Item	FM	Inclusion level (g/kg)					
		0	100	200	300	400	
Moisture (%)	72.17 ± 2.00	74.11 ± 0.57	74.48 ± 0.88	73.05 ± 0.32	72.70 ± 0.54	72.90 ± 0.36	
Crude protein (%)	17.59 ± 1.10a	16.00 ± 0.20b	16.54 ± 0.78b	15.06 ± 0.20c	14.99 ± 0.10c	15.03 ± 0.28c	
Ether extract (%)	4.98 ± 0.53b	5.43 ± 0.31b	4.40 ± 0.66b	5.95 ± 0.45a	6.47 ± 0.95a	6.17 ± 0.26a	
Ash (%)	5.20 ± 0.61	4.79 ± 0.22	4.82 ± 0.11	6.08 ± 0.15	5.62 ± 0.46	4.62 ± 0.28	
CF (%)	1.73 ± 0.04	1.73 ± 0.02	1.78 ± 0.06	1.64 ± 0.03	1.90 ± 0.15	1.72 ± 0.08	
HSI (%)	1.98 ± 0.18	2.47 ± 0.48	2.62 ± 0.16	2.90 ± 0.40	2.43 ± 0.11	3.15 ± 0.40	
VSI (%)	2.28 ± 0.27	2.35 ± 0.04	1.98 ± 0.37	2.28 ± 0.15	2.43 ± 0.38	2.22 ± 0.13	

Values are described as means ± SE. Different letters indicate significant differences (*P* < 0.05). FM: fish meal-based diet; CF: condition factor; HSI: hepatosomatic index; VSI: viscera somatic index.

### Intestinal histology

**Fig. 2:**
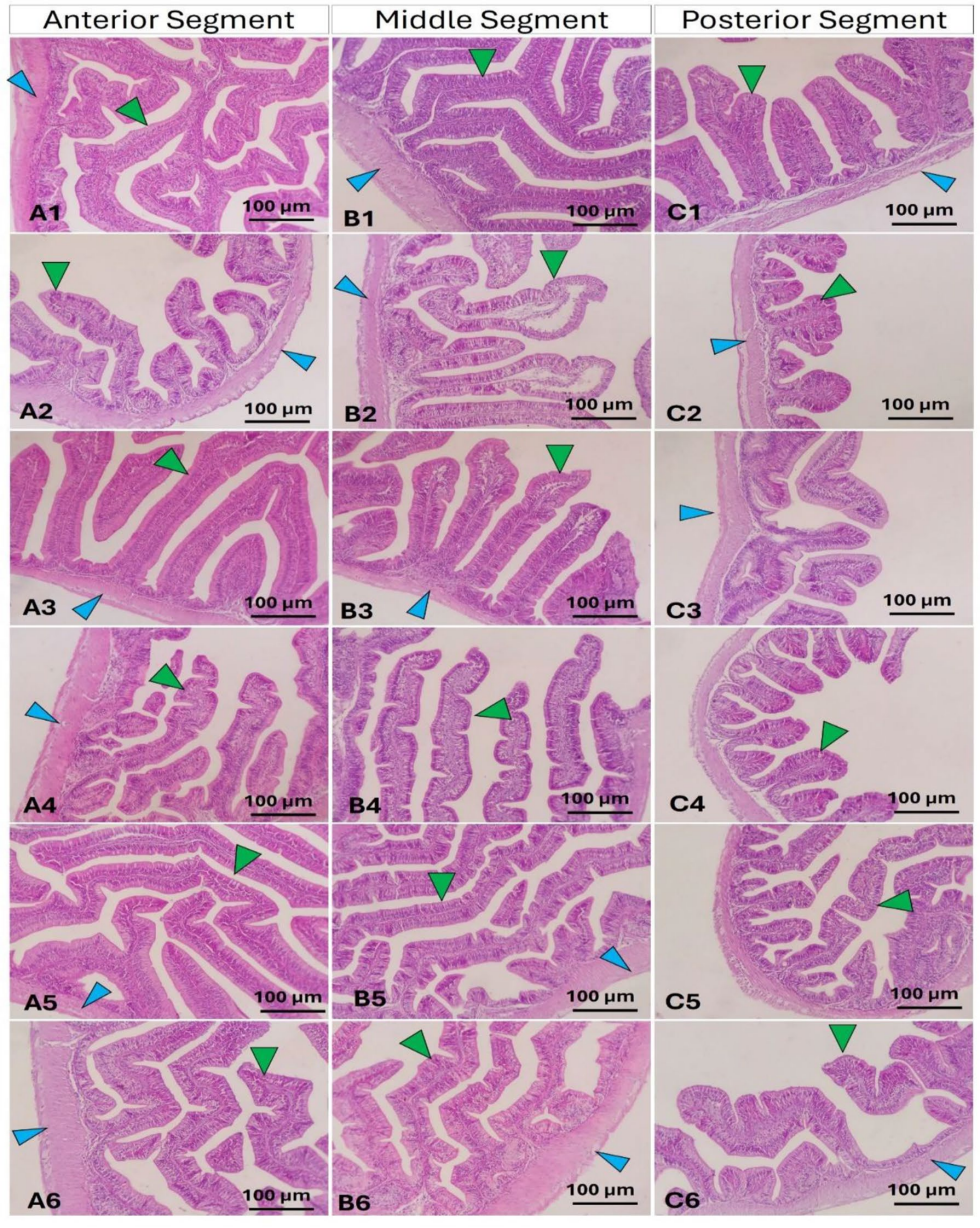
Photomicrograph of anterior, middle, and posterior segments of Nile tilapia intestine in fish fed fish meal based diet (**A1**- **C1**), a blend of oilseed meals (BOM) at 0, 100, 200, 300, and 400 g/kg levels (A-C: 2–6 respectively). The green arrowhead refers to the intestinal villi and the blue arrowhead refers to the intestinal wall. Stain H&E. Bar = 100 μm

Examination of the Nile tilapia intestine at a histological level revealed consistent structural patterns in both the intestinal mucosa and wall across all segments (anterior, middle, and posterior) in the experimental groups (Fig. 2). In the FM group (A1-C1), there was an optimal arrangement of intestinal mucosa characterized by an abundance of goblet cells in the middle segment. Conversely, fish-fed BOM at 0 g/kg (A2-C2) exhibited moderate intestinal morphology, featuring shortened intestinal villi with epithelial separation and infiltration of inflammatory cells in the submucosa of the middle segment. The groups treated with BOM at 100–200 g/kg (A-C:3,4) demonstrated considerable improvements, followed by those treated with BOM at 300 g/kg (A5-C5), while fish fed BOM at 400 g/kg (A6-C6) exhibited comparatively lesser intestinal histological features compared to the other groups.

The effects of dietary BOM on the intestinal morphometrical indices of Nile tilapia are shown in Table 5. In the anterior segment, no marked effects on the villus width (VW) and muscular thickness (MT) (*P* > 0.05). However, the villus height (VH) was markedly increased by FM and decreased by BOM at 400 g/kg (*P* < 0.05). Further, fish-fed BOM at 100, 200, and 300 g/kg showed higher VH than fish-fed BOM at 400 g/kg (*P* < 0.05). In the middle segment, fish-fed BOM at 100, 200, and 300 g/kg showed higher VH than fish-fed FM or BOM at 400 g/kg (*P* < 0.05). While the VW was markedly higher in fish-fed FM than fish fish-fed BOM (*P* < 0.05) regardless of the inclusion level. In the posterior segment, fish-fed FM or BOM at 0 g/kg showed higher VH than fish-fed BOM at 0, 100, 200, 300, and 400 g/kg (*P* < 0.05). Further fish fed BOM at 100, 200, or 300 g/kg had higher VH than fish fed BOM at 400 g/kg (*P* < 0.05).

**Table 5:** Effects of dietary blend of oilseed meals on the intestinal morphometrical indices of Nile tilapia

Item	FM	Inclusion level (g/kg)				
		0	100	200	300	400
Anterior						
Villus height (µm)	606.30 ± 17.45^a^	443.63 ± 29.48^b^	475.13 ± 55.70^b^	400.17 ± 19.50^b^	463.96 ± 10.85^b^	282.35 ± 20.82^c^
Villus width (µm)	106.96 ± 9.54	91.24 ± 10.68	113.50 ± 6.03	95.03 ± 1.70	101.66 ± 4.09	86.69 ± 3.05
Muscularis thickness (µm)	97.70 ± 5.69	101.04 ± 9.31	85.52 ± 2.22	90.28 ± 2.65	71.09 ± 4.20	65.47 ± 1.81
Middle						
Villus height (µm)	323.46 ± 11.28^b^	471.27 ± 5.33^a^	398.65 ± 17.18^a^	397.17 ± 24.82^a^	405.40 ± 4.77^a^	370.49 ± 48.74^b^
Villus width (µm)	107.24 ± 1.51^a^	77.23 ± 6.40^b^	79.49 ± 3.86^b^	77.01 ± 3.72^b^	68.89 ± 6.68^b^	63.73 ± 5.89^b^
Muscularis thickness (µm)	98.68 ± 4.70	91.08 ± 9.87	84.69 ± 3.05	67.97 ± 2.92	80.96 ± 5.34	74.80 ± 4.82
Posterior						
Villus height (µm)	308.99 ± 26.82^a^	326.15 ± 43.81^a^	264.36 ± 20.63^b^	194.20 ± 15.66^b^	284.85 ± 27.73^b^	167.61 ± 5.00^c^
Villus width (µm)	87.62 ± 5.50	87.00 ± 2.84	90.73 ± 1.41	80.73 ± 1.18	76.33 ± 5.81	94.23 ± 6.48
Muscularis thickness (µm)	53.16 ± 1.14	71.68 ± 4.98	55.11 ± 6.01	51.08 ± 2.84	68.70 ± 6.27	68.42 ± 1.31

Values are described as means ± SE. Different letters indicate significant differences (*P* < 0.05). FM: fish meal-based diet.

### Blood biochemistry

The effects of dietary BOM on the blood biochemical traits of Nile tilapia are shown in Table 6. No marked effects on the ALT, AST, total protein, albumin, globulin, urea, and creatinine (*P* > 0.05).

**Table 6:** Effects of dietary blend of oilseed meals on the blood biochemistry indices of Nile tilapia

Item	FM	Inclusion level (g/kg)				
		0	100	200	300	400
ALT (U/I)	16.67 ± 1.67	16.00 ± 4.93	15.00 ± 0.00	16.00 ± 1.00	16.67 ± 1.76	16.67 ± 1.67
AST (U/I)	6.01 ± 0.65	6.00 ± 0.53	5.43 ± 1.15	5.00 ± 1.53	6.33 ± 0.67	6.00 ± 0.58
Total protein (g/dl)	4.20 ± 0.25	4.13 ± 0.09	4.23 ± 0.15	4.63 ± 0.12	4.47 ± 0.15	4.40 ± 0.15
Albumin (g/dl)	1.02 ± 0.10	0.98 ± 0.04	1.03 ± 0.05	1.12 ± 0.10	1.23 ± 0.15	1.11 ± 0.04
Globulin (g/dl)	3.18 ± 0.22	3.16 ± 0.12	3.20 ± 0.11	3.51 ± 0.18	3.23 ± 0.25	3.29 ± 0.14
Urea (mg/dl)	7.33 ± 0.67	7.67 ± 0.33	6.33 ± 0.67	7.33 ± 0.88	7.67 ± 0.33	7.33 ± 0.67
Creatinine (mg/dl)	0.50 ± 0.05	0.55 ± 0.08	0.40 ± 0.01	0.40 ± 0.02	0.46 ± 0.02	0.43 ± 0.01

Values are described as means ± SE. Different letters indicate significant differences (*P* < 0.05). FM: fish meal-based diet; ALT: alanine aminotransferase, AST: aspartate aminotransferase.

### Gene expression

**Fig. 3:**
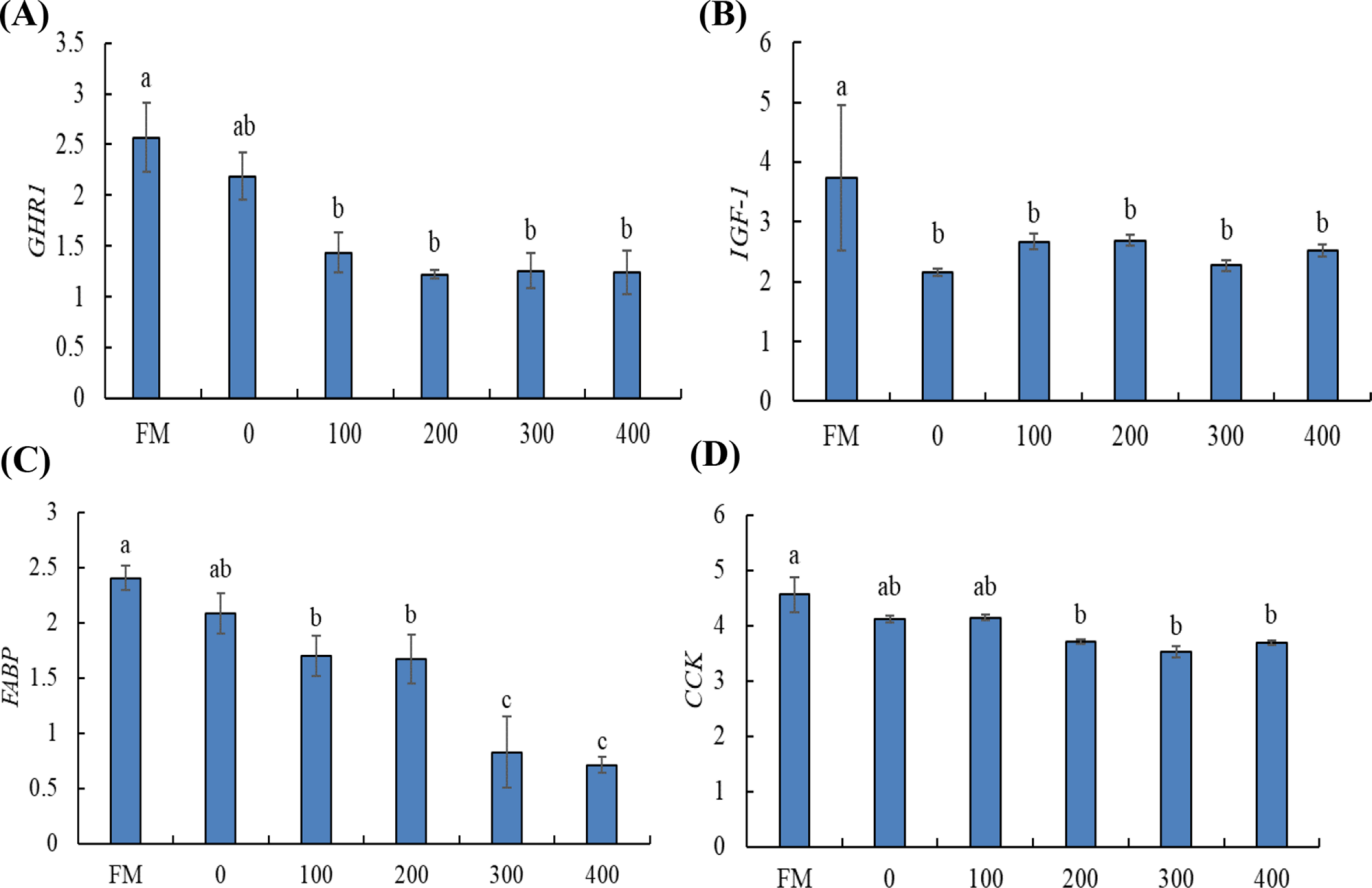
Effects of dietary blend of oilseed meals on the relative expression of growth and metabolic genes (**A**) *GHR1*, (**B**) *IGF-1*, (**C**) *FABP*, and (**D**) *CCK* in Nile tilapia. Values are described as means ± SE. Different letters indicate significant differences (*P* < 0.05). FM: fish meal-based diet

The effects of dietary BOM on the relative expression of growth and metabolic genes (*GHR1*, *IGF-1*, *FABP*, and *CCK*) in Nile tilapia are shown in Fig. 3. The relative expression of the *GHR1* gene was downregulated in tilapia-fed BOM at 100, 200, 300, and 400 g/kg compared to fish-fed FM-based diet (*P* < 0.05) and without differences with fish-fed SBM-based diet (*P* > 0.05) (Fig. 3A). The3044 relative expression of the *IGF-1* gene was downregulated in tilapia-fed BOM at 0, 100, 200, 300, and 400 g/kg compared to fish-fed FM-based diet (*P* < 0.05) (Fig. 3B). The relative expression of the *FABP* gene was downregulated in tilapia-fed BOM at 100, 200, 300, and 400 g/kg compared to fish-fed FM-based diet (*P* < 0.05) (Fig. 3C). Further, tilapia fed 300 and 400 g/kg showed the lowest relative expression of *FABP* compared to the other groups (*P* < 0.05). Tilapia-fed SBM-based diet showed no significant differences with fish FM-based diet (*P* > 0.05), 100, and 200 g BOM/kg. The relative expression of the *CCK* gene was downregulated in tilapia-fed BOM at 200, 300, and 400 g/kg compared to fish-fed FM-based diet (*P* < 0.05) and without differences with fish fed 0 and 100 g/kg (*P* > 0.05) (Fig. 3D).

### Economic evaluation

The economic evaluation of dietary BOM in the diets of Nile tilapia is shown in Table 7. The cost of feed needed for 1 kg fish fed FM, SBM, or BOM at 100, 200, 300, and 400 g/kg is 0.74, 0.67, 0.65, 0.65, 0.66, and 0.64 USD/kg weight gain, respectively. The relative cost of feed per kg fish gain showed 4.42, 7.11, 8.14, 10.32, and 8.10% reduction rates in fish-fed SBM, or BOM at 100, 200, 300, and 400 g/kg compared to fish-fed FM-based diet.

**Table 7:** Economic evaluation of dietary blend of oilseed meals in the diets of Nile tilapia

Items	FM	Inclusion level (g/kg)				
		0	100	200	300	400
Feed cost (USD/kg)	0.53	0.50	0.48	0.48	0.46	0.45
FCR	1.39	1.33	1.35	1.37	1.43	1.44
Cost of feed for 1 kg fish (USD/kg weight gain)	0.74	0.67	0.65	0.65	0.66	0.64
Cost reduction of feed per 1 kg fish (USD/kg weight gain)	0.00	0.03	0.05	0.05	0.07	0.05
Relative cost reduction of feed per kg fish gain (%)	0.00	4.42	7.11	8.14	10.32	8.10

FCR: feed conversion ratio

## Discussion

Tilapia farming is growing globally due to its high food quality and tolerance to biotic and abiotic stressors [67]. Nevertheless, the high cost of feed ingredients is limiting the feasibility and challenging the sustainability of tilapia farming [68]. Fish meal (FM) and soybean meal (SBM) are the main components of protein content required for nutritionally balanced aquafeed production [69]. Due to the decreasing availability and high cost of FM and SBM, huge efforts have investigated multiple protein alternatives in tilapia feeds [70]. More specifically, substituting FM with alternative animal or plant protein sources [22, 23]. Tilapia is an omnivorous fish species that can utilize animal and plant protein sources [18]. Dietary SBM is another high-value protein content but causes another challenge for tilapia production [67, 71]. In this context, many efforts have been conducted on the possibility of replacing SBM with alternative plant protein sources. Plant byproducts and oilseed meals are the most utilized ingredients to replace SBM such as cottonseed meal (CSM) [30], linseed meal (LSM) [42], sesame meal (SSM) [35], and sunflower meal (SFM) [39]. In most cases, SBM was replaced totally or partially with one of the above-mentioned ingredients. Synergistic mixtures of plant ingredients are nutritionally balanced sources of protein that can replace FM or SBM in aquafeed [72, 73]. The individual replacement of CSM, LSM, SSM, and SFM could lead to a high presence of anti-nutritional factors (ANFs) and an imbalance of essential amino acid content [74, 75]. Thus the blend of these ingredients could result in compensation of limited amino acids compared to the individual inclusion of these ingredients. In the present study, a blend of oilseed meals (BOM) containing CSM + LSM + SSM + SFM at the rate of 1:1:1:1 was used to partially substitute SBM in Nile tilapia diets.

The results of this study indicate that dietary BOM can be included in Nile tilapia diets up to 129.75 g/kg according to the results of the regression analysis. The inclusion level of BOM was based on reducing the level of SBM in FM-free diets without interrupting the final body weight. Compared to FM based diet, no marked effects were observed on the FBW, SGR, and feed intake. While Nile tilapia fed dietary BOM at 0, 100, and 200 g/kg showed higher weight gain (WG) and protein efficiency ration (PER) than those fed FM or BOM at 300 and 400 g/kg. These results are in line with earlier reports that indicate the ability of Nile tilapia to feed on CSM [30], LSM [42], SSM [35], and SFM [39] as replacers for FM or SBM without affecting the growth performance. The high inclusion levels of BOM led to a reduction in the WG which can be possibly due to the presence of ANFs [74]. The results also indicated that feed conversion ratio (FCR) showed a marked reduction by FM and BOM at 100–200 g/kg compared to fish-fed BOM at 300 and 400 g/kg. According to the results of one-way ANOVA, the results indicated that Nile tilapia can utilize BOM up to 200 g/kg without affecting the the growth performance and feed utilization. These results match with earlier efforts that validated the possible substitution of FM or SBM with CSM, LSM, SSM, and SFM ingredients up to certain levels. In this regard, dietary CSM, SSM, and SFM ingredients can be included in Nile tilapia diets up to 90 g/kg [30], 84.7 g/kg [34], and 64.75 g/kg [39], respectively. The mixture of CSM, LSM, SSM, and SFM could enrich feed formulations with supplementary nutrients required for suitable feed utilization and metabolic function compared to individual usage [72, 73, 76]. Nonetheless, high inclusion levels of BOM negatively impacted growth performance (WG) and feed utilization (FCR and PER). Dietary CSM, LSM, SSM, and SFM ingredients contain abundant ANFs such as (gossypol, glucosinolate, and tannin) involved in the reduction of palatability of feeds thereby reducing feed utilization (FCR and PER) [77, 78]. In addition, the high crude fiber content in CSM, LSM, SSM, and SFM ingredients could reduce feed utilization and absorption [74]. Although feed intake did not markedly differ among the groups of fish fed FM, SBM, or BOM-based diets the reduction of PER and aise of FCR by 300 and 400 g/kg BOM could be related to low feed utilization in fish intestines. Similarly, W-J Li, H-X Wu, L Zhang, M Li, T Wang, C-J Shan, F Qiao, L-Q Chen, W-B Zhang, Z-Y Du, et al. [30], O Olude, F George and W Alegbeleye [35], and EO Ogello, EM Kembenya, CM Githukia, CN Aera, JM Munguti and CS Nyamweya [39] reported reduced feed utilization in Nile tilapia-fed CSM, SSM, and SFM, respectively. It has been reported that high fiber content could decrease the digestibility and increase the dilution of nutrients thereby lowering absorption and feed utilization [79].

The study tested the effects of BOM inclusion on the carcass composition and somatic index. No marked effects on the carcass composition contents of moisture and ash contents while crude protein content was markedly increased by FM and BOM at 0–100 g/kg. These results are in line with MS Hassaan, AIM El-Sayed, MA Soltan, MM Iraqi, AM Goda, SJ Davies, ER El-Haroun and HA Ramadan [29] and Y-X Guo, X-H Dong, B-P Tan, S-Y Chi, Q-H Yang, G Chen and L Zhang [34] who stated that dietary CSM and SSM did not affect the moisture and ash contents in the body composition of Nile tilapia. While MS Hassaan, AIM El-Sayed, MA Soltan, MM Iraqi, AM Goda, SJ Davies, ER El-Haroun and HA Ramadan [29] and Y-X Guo, X-H Dong, B-P Tan, S-Y Chi, Q-H Yang, G Chen and L Zhang [34] reported that dietary CSM and SSM increased the protein content in the carcass of Nile tilapia. These results illustrate that dietary BOM up to 100 g/kg is well utilized by fish during protein synthesis resulting in protein nutrient availability for efficient muscle growth and metabolic function. Furthermore, the ether extract content showed marked improvement in fish BOM at 200, 300, and 400 g/kg. Increased ether extract content can be related to the presence of lipids in BOM when included at a high ratio since BOM is rich in oil derivatives resulting during extraction [25, 26]. The results also indicated no marked effects on the somatic indices including condition factor, hepatosomatic index, and viscerasomatic index of Nile tilapia-fed BOM. The results of the somatic index confirm that dietary BOM has no negative effects on reserved energy relative to the nutritional status.

The assessment of the intestinal histological structure is a vital tool involved in the evaluation of the effects of novel feed formulations on intestinal health [80]. The health of fish intestines is the first key factor that guarantees efficient digestion, metabolic, and physiological function thereby healthy and productive fish [81]. On the other hand, the correlation between growth performance data and improvements in histology measures is an important component of understanding fish development; nevertheless, it is crucial to note that a perfect relationship between these two parameters is not always required [82, 83]. A variety of variables influence fish growth and development, including environmental circumstances, diet, handling, and stress levels, all of which have varied effects on growth rates and histology results [82, 84]. For example, whereas improved feeding techniques may result in increased growth performance, the related histological alterations may not necessarily represent this growth owing of differences in metabolic responses or adaptations to varied feeding regimens [85]. The results indicated uniform structural patterns in both the intestinal mucosa and wall across all segments (anterior, middle, and posterior) in the experimental groups. In the FM group, there was an optimal arrangement of intestinal mucosa characterized by an abundance of goblet cells in the middle segment. Conversely, fish-fed BOM exhibited moderate intestinal morphology, featuring shortened intestinal villi with epithelial separation and infiltration of inflammatory cells in the submucosa of the middle segment in a dose-dependent manner. The groups treated with BOM at 100–200 g/kg demonstrated considerable impairments, followed by those treated with BOM at 300 g/kg, while fish fed BOM at 400 g/kg exhibited comparatively lesser intestinal histological features compared to the other groups. The results are in line with, W-J Li, H-X Wu, L Zhang, M Li, T Wang, C-J Shan, F Qiao, L-Q Chen, W-B Zhang, Z-Y Du, et al. [30] who reported that the high inclusion levels of dietary CSM could impair the intestinal histological features in Nile tilapia. Concurrently, the reduction of growth performance and feed utilization in the Nile tilapia-fed BOM at high inclusion levels (300 and 400 g/kg) can be explained by the negative impacts of BOM on the intestines. Indeed the reduction of intestinal villi height and width could lead to low surface area required for feed digestion and absorption thereby low feed utilization [86].

Blood biochemical traits are suitable tools to evaluate the metabolic and physiological conditions of fish [87]. Specifically when incorporating alternative protein ingredients in fish feeds. In this study, dietary BOM showed no marked effects on the ALT, AST, total protein, albumin, globulin, urea, and creatinine traits of Nile tilapia. Similarly, CP Duodu, D Adjei-Boateng, AK Amponsah, P Andrews and KA Obirikorang [76] stated that dietary plant protein-based diets did not compromise the blood biochemical traits in Nile tilapia. The ALT and AST activities refer to the liver function and are involved in the amino acid transfer and their high release in the bloodstream indicates liver function failure [88, 89]. Therefore, the absence of differences among the groups of fish-fed FM, SBM, or BOM-based diets indicates healthy liver function and safe use for BOM in tilapia feeding. It cannot be ignored also that the kidney function-related bioindicators (urea and creatinine) in Nile tilapia-fed FM, SBM, or BOM-based diets showed no marked differences among the groups. Alongside, the total protein globulin, and albumin were not markedly affected by dietary BOM inclusion in tilapia diets. These results confirm that dietary BOM has no negative impact on kidney function under the current trial condition.

Fish growth is closely correlated with the expression of growth and metabolic genes [90]. To explain the mode of action for dietary BOM on the growth performance and feed utilization we tested the relative expression of *GHR1*, *IGF-1*, *FABP*, and *CCK* genes. *GHR1* and *IGF-1* genes are growth-related mediators involved in the release of growth hormone and their expression can be affected by the feed composition and nutritional status of fish [91]. Further *FABP* gene is involved in regulating lipid metabolism and energy initiation required for proper growth performance and well-being of fish [92, 93]. While the *CCK* gene regulates the digestive enzyme activity leading to efficient feed digestion and absorption [94, 95]. The results of this study indicated that the relative expression of *GHR1*, *IGF-1*, *FABP*, and *CCK* genes were downregulated in tilapia-fed BOM compared to fish-fed FM-based diet with the lowest transcription value in fish fed 400 g BOM/kg. The results are in line with MS Hassaan, AIM El-Sayed, MA Soltan, MM Iraqi, AM Goda, SJ Davies, ER El-Haroun and HA Ramadan [29] who reported that dietary CSM could downregulate the expression of *GHR1* and *IGF-1* genes in Nile tilapia. These results indicate that the reduction of feed utilization and growth performance of Nile tilapia fed with high inclusion levels of BOM can be attributed to the downregulation of *GHR1*, *IGF-1*, *FABP*, and *CCK* genes. Downregulation of both *FABP* and *CCK* by high levels of BOM indicates that lowered growth performance can be related to the reduction of lipid metabolism and digestive enzyme activity [95]. The reduction of *GHR1*, *IGF-1*, *FABP*, and *CCK* genes in Nile tilapia under the current study conditions can be related to the presence of ANFs and crude fibers which are well known for their negative impacts on the feed metabolism [96]. In this regard, M Samtiya, RE Aluko and T Dhewa [97] reported that ANF-rich nutrients could reduce feed metabolism via binding with vitamins, minerals, and proteins thereby decreasing the absorption in the intestines. Further, ANFs contain several components such as phytate, tannins, lectin, trypsin inhibitor, amylase, saponins, and oxalate which impact feed metabolism by harming protein digestibility by protein inhibitors, hampering amino acid bioavailability by tannins, impairment of hydrolytic and transport functions at the enterocyte site by lectins [74]. Therefore elimination of ANFs through some processing techniques has been applied to enhance the quality of plant protein-sourced ingredients [77, 78].

The economic evaluation of dietary BOM showed a reduction in the production cost of Nile tilapia compared to the positive control group (FM). These results indicate that using BOM could enhance the feasibility of Nile tilapia production but further future studies are suggested to utilize processed BOM in Nile tilapia nutrition. Besides, relevant feed additives such as attractants, probiotics, exogenous digestive enzymes, and growth promoters are suggested to enhance the utilization of BOM in Nile tilapia diets [98, 99].

## Conclusion

The blend of oilseed meals (cottonseed meal, linseed meal, sesame meal, and sunflower meal) can be included in the soybean based-diets of Nile tilapia up to 200 g/kg without hampering the growth performance and feed utilization. High inclusion levels (300 and 400 g/kg) could negatively affect the growth performance and feed utilization by interrupting the intestinal histological features and suppressing of expression of growth and metabolic genes (*GHR1*, *IGF-1*, *FABP*, and *CCK*) in the liver. Therefore, further future studies are suggested to investigate possible strategies for increasing the inclusion level of dietary blends of oilseed meals to have sustainable and feasible Nile tilapia production.

## Data Availability

Data is provided within the manuscript and are available upon request from the corresponding author.

## References

[1] Bjørndal T, Dey M, Tusvik A. Economic analysis of the contributions of aquaculture to future food security. Aquaculture. 2024;578:740071.

[2] Dewali S, Sharma N, Melkani D, Arya M, Kathayat N, Panda AK, Bisht SS. Aquaculture: Contributions to Global Food Security. In: *Emerging Solutions in Sustainable Food and Nutrition Security.* edn. Edited by Ghosh S, Kumari Panda A, Jung C, Singh Bisht S. Cham: Springer International Publishing; 2023: 123–139.

[3] Subasinghe R, Soto D, Jia J. Global aquaculture and its role in sustainable development. Reviews Aquaculture. 2009;1(1):2–9.

[4] Varzakas T, Smaoui S. Global Food Security and Sustainability issues: the Road to 2030 from Nutrition and sustainable healthy diets to Food systems Change. In: Foods vol. 13; 2024. 10.3390/foods13020306 PMC10815419 38254606

[5] Gatlin Iii DM, Barrows FT, Brown P, Dabrowski K, Gaylord TG, Hardy RW, Herman E, Hu G, Krogdahl Å, Nelson R, et al. Expanding the utilization of sustainable plant products in aquafeeds: a review. Aquac Res. 2007;38(6):551–79.

[6] Auchterlonie NA. Fishmeal and fish oil in aquaculture feeds: global food security questions. Int J Environ Stud. 2024;81(2):896–913.

[7] Hussain SM, Bano AA, Ali S, Rizwan M, Adrees M, Zahoor AF, Sarker PK, Hussain M, Arsalan MZ-u-H, Yong JWH et al. Substitution of fishmeal: Highlights of potential plant protein sources for aquaculture sustainability. *Heliyon* 2024, 10(4). 10.1016/j.heliyon.2024.e26573 PMC10906437 38434023

[8] Muntean G-C, Simedru D, Uiuiu P, Tanaselia C, Cadar O, Becze A, Coroian A. Evaluation of alternative sources of proteins and other nutrients with potential applications in Fish Nutrition. In: Molecules 29; 2024. 10.3390/molecules29102332 PMC11123814 38792193

[9] Gasco L, Gai F, Maricchiolo G, Genovese L, Ragonese S, Bottari T, Caruso G. Fishmeal Alternative Protein Sources for Aquaculture Feeds. In: *Feeds for the Aquaculture Sector: Current Situation and Alternative Sources.* edn. Edited by Gasco L, Gai F, Maricchiolo G, Genovese L, Ragonese S, Bottari T, Caruso G. Cham: Springer International Publishing; 2018: 1–28.

[10] Macusi ED, Cayacay MA, Borazon EQ, Sales AC, Habib A, Fadli N, Santos MD. Protein fishmeal replacement in aquaculture: a systematic review and implications on growth and adoption viability. In: Sustainability 15; 2023.

[11] Hussain SM, Bano AA, Ali S, Rizwan M, Adrees M, Zahoor AF, Sarker PK, Hussain M, Arsalan MZ-u-H, Yong JWH, et al. Substitution of fishmeal: highlights of potential plant protein sources for aquaculture sustainability. Heliyon. 2024;10(4):e26573. 10.1016/j.heliyon.2024.e26573 PMC10906437 38434023

[12] Abdel-Hady MM, El-karashily AF, Salem AM, Haggag SM. Sustainable fish production in Egypt: towards strategic management for capture-based aquaculture. Aquacult Int 2024.

[13] FAO: World Fisheries and Aquaculture. Food and Agriculture Organization, Rome. (2022), 10.4060/cc0461en. 2022.

[14] Furuya WM, Cruz TP, Gatlin DM. Amino acid requirements for Nile Tilapia: an update. In: Animals vol. 13; 2023. 10.3390/ani13050900 PMC10000143 36899757

[15] Arumugam M, Jayaraman S, Sridhar A, Venkatasamy V, Brown PB, Abdul Kari Z, Tellez-Isaias G, Ramasamy T. Recent advances in Tilapia Production for sustainable developments in Indian aquaculture and its economic benefits. In: Fishes vol. 8; 2023.

[16] El-Sayed A-FM, Dickson MW, El-Naggar GO. Value chain analysis of the aquaculture feed sector in Egypt. Aquaculture. 2015;437:92–101.

[17] Temesgen M, Getahun A, Lemma B, Janssens GPJ. Food and Feeding Biology of Nile Tilapia (*Oreochromis niloticus*) in Lake Langeno, Ethiopia. In: Sustainability 14; 2022.

[18] Canonico GC, Arthington A, McCrary JK, Thieme ML. The effects of introduced tilapias on native biodiversity. Aquat Conservation: Mar Freshw Ecosyst. 2005;15(5):463–83.

[19] El-Sayed A-FM, Nasr-Allah AM, Dickson M, Gilmour C. Analysis of aquafeed sector competitiveness in Egypt. Aquaculture. 2022;547:737486.

[20] Ahmadifar E, Pourmohammadi Fallah H, Yousefi M, Dawood MAO, Hoseinifar SH, Adineh H, Yilmaz S, Paolucci M, Doan HV. The Gene Regulatory Roles of Herbal Extracts on the Growth, Immune System, and Reproduction of Fish. *Animals (Basel)* 2021, 11(8). 10.3390/ani11082167 PMC8388479 34438625

[21] Ahmadifar E, Mohammadzadeh S, Kalhor N, Yousefi M, Moghadam MS, Naraballobh W, Ahmadifar M, Hoseinifar SH, Van Doan H. Cornelian cherry (Cornus mas L.) fruit extract improves growth performance, disease resistance, and serum immune-and antioxidant-related gene expression of common carp (Cyprinus carpio). Aquaculture. 2022;558:738372.

[22] Jia S, Li X, He W, Wu G. Protein-Sourced Feedstuffs for Aquatic Animals in Nutrition Researchand Aquaculture. In: *Recent Advances in Animal Nutrition and Metabolism.* edn. Edited by Wu G. Cham: Springer International Publishing; 2022: 237–261. 10.1007/978-3-030-85686-1_12 34807445

[23] Pailan GH, Biswas G. Feed and feeding strategies in Freshwater Aquaculture. Transforming Coastal Zone for sustainable food and income security: 2022// 2022; Cham. Springer International Publishing; 2022. pp. 455–75.

[24] Hoseinifar SH, Khalili M, Rufchaei R, Raeisi M, Attar M, Cordero H, Esteban MÁ. Effects of date palm fruit extracts on skin mucosal immunity, immune related genes expression and growth performance of common carp (Cyprinus carpio) fry. Fish Shellfish Immunol. 2015;47(2):706–11. 26439417 10.1016/j.fsi.2015.09.046

[25] Kumar M, Kumari N, Prakash S, Sharma N, Radha, Sharma K, Chandran D, Raman P, Panesar PS. Cottonseed Meal: Eliminating Gossypol for Securing Another Source of Protein. In: *Oilseed Meal as a Sustainable Contributor to Plant-Based Protein: Paving the Way Towards Circular Economy and Nutritional Security.* edn. Edited by Kumar M, Punia Bangar S, Panesar PS. Cham: Springer International Publishing; 2024: 145–167.

[26] Li MH, Robinson EH. Use of Cottonseed Meal in aquatic animal diets: a review. North Am J Aquaculture. 2006;68(1):14–22.

[27] Mbahinzireki D, Lee ES, Wisner. Growth, feed utilization and body composition of tilapia (*Oreochromis* sp.) fed with cottonseed meal-based diets in a recirculating system. Aquacult Nutr. 2001;7(3):189–200.

[28] El-Saidy DMSD, Gaber MM. Use of cottonseed meal supplemented with iron for detoxification of gossypol as a total replacement of fish meal in Nile tilapia, *Oreochromis niloticus* (L.) diets. Aquac Res. 2004;35(9):859–65.

[29] Hassaan MS, El-Sayed AIM, Soltan MA, Iraqi MM, Goda AM, Davies SJ, El-Haroun ER, Ramadan HA. Partial dietary fish meal replacement with cotton seed meal and supplementation with exogenous protease alters growth, feed performance, hematological indices and associated gene expression markers (GH, IGF-I) for Nile tilapia, *Oreochromis niloticus*. Aquaculture. 2019;503:282–92.

[30] Li W-J, Wu H-X, Zhang L, Li M, Wang T, Shan C-J, Qiao F, Chen L-Q, Zhang W-B, Du Z-Y, et al. Effects of replacing soybean meal protein with cottonseed protein concentrate on the growth condition and intestinal health of Nile tilapia (*Oreochromis niloticus*). Aquacult Nutr. 2021;27(6):2436–47.

[31] Abbas S, Sharif MK, Sibt-e-Abbas M, Fikre Teferra T, Sultan MT, Anwar MJ. Nutritional and therapeutic potential of Sesame seeds. J Food Qual. 2022;2022(1):6163753.

[32] Wei P, Zhao F, Wang Z, Wang Q, Chai X, Hou G, Meng Q. Sesame (*Sesamum indicum* L.): a Comprehensive Review of Nutritional Value, Phytochemical Composition, Health benefits, development of Food, and Industrial Applications. In: Nutrients 14; 2022. 10.3390/nu14194079 PMC9573514 36235731

[33] Wacal C, Musinguzi SP, Ewaju E, Atibo C, Alowo D, Alipa J, Basalirwa D. Unravelling the potential benefits of sesame (*Sesamum indicum* L.) in cropping systems, nutritional, health, and industrial uses of its seeds – a review. Cogent Food Agric. 2024;10(1):2360766.

[34] Guo Y-X, Dong X-H, Tan B-P, Chi S-Y, Yang Q-H, Chen G, Zhang L. Partial replacement of soybean meal by sesame meal in diets of juvenile Nile tilapia, *Oreochromis niloticus* L. Aquac Res. 2011;42(9):1298–307.

[35] Olude O, George F, Alegbeleye W. Utilization of autoclaved and fermented sesame (*Sesamum indicum* L.) seed meal in diets for Til-aqua natural male tilapia. Anim Nutr. 2016;2(4):339–44. 29767031 10.1016/j.aninu.2016.09.001 PMC5941054

[36] Lannuzel C, Smith A, Mary AL, Della Pia EA, Kabel MA, de Vries S. Improving fiber utilization from rapeseed and sunflower seed meals to substitute soybean meal in pig and chicken diets: a review. Anim Feed Sci Technol. 2022;285:115213.

[37] Ditta YA, King AJ. Recent advances in sunflower seed meal as an alternate source of protein in broilers. World’s Poult Sci J. 2017;73(3):527–42.

[38] Hadidi M, Aghababaei F, McClements DJ. Sunflower meal/cake as a sustainable protein source for global food demand: towards a zero-hunger world. Food Hydrocolloids. 2024;147:109329.

[39] Ogello EO, Kembenya EM, Githukia CM, Aera CN, Munguti JM, Nyamweya CS. Substitution of fish meal with sunflower seed meal in diets for Nile tilapia (*Oreochromis niloticus* L.) reared in earthen ponds. J Appl Aquac. 2017;29(1):81–99.

[40] Hassaan MS, Soltan MA, Mohammady EY, Elashry MA, El-Haroun ER, Davies SJ. Growth and physiological responses of Nile tilapia, *Oreochromis niloticus* fed dietary fermented sunflower meal inoculated with *Saccharomyces cerevisiae* and *Bacillus subtilis*. Aquaculture. 2018;495(November 2017):592–601.

[41] Cloutier S. Linseed: Overview. In: *Encyclopedia of Food Grains (Second Edition).* edn. Edited by Wrigley C, Corke H, Seetharaman K, Faubion J. Oxford: Academic Press; 2016: 259–264.

[42] Pianesso D, Goulart FR, Adorian TJ, Mombach PI, de Lima JS, dos Santos TS, da Silva LP. Linseed protein concentrate as alternative to fishmeal in diets for silver catfish, *Rhamdia quelen*. J World Aquaculture Soc. 2021;52(2):316–28.

[43] Soltan NM, Soaudy MR, Abdella MM, Hassaan MS. Partial dietary fishmeal replacement with mixture of plant protein sources supplemented with exogenous enzymes modify growth performance, digestibility, intestinal morphology, haemato-biochemical and immune responses for Nile tilapia, *Oreochromis niloticus*. Anim Feed Sci Technol. 2023;299:115642.

[44] El-Saidy DMSD, Gaber MMA. Replacement of fish meal with a mixture of different plant protein sources in juvenile Nile tilapia, *Oreochromis niloticus* (L.) diets. Aquac Res. 2003;34(13):1119–27.

[45] Mugwanya M, Dawood MAO, Kimera F, Sewilam H. A meta-analysis on the influence of dietary betaine on the growth performance and feed utilization in aquatic animals. Aquaculture Rep. 2024;37:102200.

[46] AOAC: International. Official Methods of Analysis of AOAC International. 20th ed. Gaithersburg, MD, USA: AOAC International. 2016:3172.

[47] El-Desouky FF, Ibrahim MA, Abd El-Razek IM, El-Nabawy E-SM, Amer AA, Zaineldin AI, Gewaily MS, Dawood MAO. Improving yellow mealworm (*Tenebrio molitor*) utilization with Sodium Butyrate in Nile Tilapia diets: effects on Growth Performance, Intestinal Histology, Antioxidative Response, and blood biomarkers. Aquacult Nutr. 2024;2024(1):2442308. 10.1155/2024/2442308 PMC11003378 39555554

[48] Kim K-W, Kim K-D, Han HS, Moniruzzaman M, Yun H, Lee S, Bai SC. Optimum dietary protein level and protein-to-energy ratio for growth of juvenile parrot fish, Oplegnathus fasciatus. J World Aquaculture Soc. 2017;48(3):467–77.

[49] Cannon D. I O, JA I: Proteins in clinical chemistry: principles and technics. NewYork: Harper & Row,John Weatherhill; 1974.

[50] Doumas BT, Bayse DD, Carter RJ, Peters T, Schaffer R. A candidate reference method for determination of total protein in serum. I. Development and validation. Clin Chem. 1981;27(10):1642–50. 6169466

[51] Reitman S, Frankel S. A colorimetric method for the determination of serum glutamic oxalacetic and glutamic pyruvic transaminases. Am J Clin Pathol. 1957;28(1):56–63. 13458125 10.1093/ajcp/28.1.56

[52] Bartles H, Bohmer M, Heirli C. Colorimetric kinetic method for creatinine determination in serum and urine. Clin Chem Acta. 1972;37:193–5. 10.1016/0009-8981(72)90432-9 5022083

[53] Fawcett J, Scott J. A rapid and precise method for the determination of urea. J Clin Pathol. 1960;13(2):156–9. 13821779 10.1136/jcp.13.2.156 PMC480024

[54] Bancroft J, Stevens A, Turner D. Theory and practice of histological techniques: Churchill Livingstone New York. the text. 766. 1996.

[55] Gewaily MS, Abumandour MM. Gross morphological, histological and scanning electron specifications of the oropharyngeal cavity of the hooded crow (Corvus cornix pallescens). Anat Histol Embryol. 2021;50(1):72–83. 32794280 10.1111/ahe.12602

[56] Schneider CA, Rasband WS, Eliceiri KW. NIH Image to ImageJ: 25 years of image analysis. Nat Methods. 2012;9(7):671–5. 22930834 10.1038/nmeth.2089 PMC5554542

[57] El-Kassas S, Aljahdali N, Abdo SE, Alaryani FS, Moustafa EM, Mohamed R, Abosheashaa W, Abdulraouf E, Helal MA, Shafi ME et al. Moringa oleifera Leaf Powder Dietary inclusion differentially modulates the antioxidant, inflammatory, and histopathological responses of normal and Aeromonas hydrophila-infected mono-sex Nile Tilapia (*Oreochromis niloticus*). Front Vet Sci 2022, 9. 10.3389/fvets.2022.918933 PMC9260175 35812877

[58] Livak KJ, Schmittgen TD. Analysis of relative gene expression data using real-time quantitative PCR and the 2^–∆∆CT^ method. Methods. 2001;25(4):402–8. 11846609 10.1006/meth.2001.1262

[59] Con P, Nitzan T, Slosman T, Harpaz S, Cnaani A. Peptide transporters in the primary gastrointestinal tract of pre-feeding Mozambique Tilapia Larva. Front Physiol 2019, 10(808). 10.3389/fphys.2019.00808 PMC6624445 31333482

[60] El-Naggar K, Mohamed R, El-katcha MI, Abdo SE, Soltan MA. Plant Ingredient diet supplemented with lecithin as fish meal and fish oil alternative affects growth performance, serum biochemical, lipid metabolism and growth-related gene expression in Nile tilapia. *Aquacu Res* 2021, n/a(n/a).

[61] El-Kassas S, Abdo SE, Abosheashaa W, Mohamed R, Moustafa EM, Helal MA, El-Naggar K. Growth performance, serum lipid profile, intestinal morphometry, and growth and lipid indicator gene expression analysis of mono-sex Nile tilapia fed Moringa oleifera leaf powder. Aquace Rep. 2020;18:100422.

[62] Zhang X, Zhong H, Han Z, Tang Z, Xiao J, Guo Z, Wang F, Luo Y, Zhou Y. Effects of waterborne exposure to 17β-estradiol on hepatic lipid metabolism genes in tilapia (*Oreochromis niloticus*). Aquac Rep. 2020;17:100382.

[63] Zou Q, Huang Y, Cao J, Zhao H, Wang G, Li Y, Pan Q. Effects of four feeding stimulants in high plant-based diets on feed intake, growth performance, serum biochemical parameters, digestive enzyme activities and appetite-related genes expression of juvenile GIFT tilapia (Oreochromis Sp). Aquacult Nutr 2017, 23.

[64] Abdel-Tawwab M, Khalil RH, Metwally AA, Shakweer MS, Khallaf MA, Abdel-Latif HMR. Effects of black soldier fly (*Hermetia illucens* L.) larvae meal on growth performance, organs-somatic indices, body composition, and hemato-biochemical variables of European sea bass, *Dicentrarchus labrax*. Aquaculture. 2020;522:735136.

[65] Zaineldin AI, Hegazi S, Koshio S, Ishikawa M, El Basuini MF, Dossou S, Dawood MAO. The influences of Bacillus subtilis C-3102 inclusion in the red sea bream diet containing high levels of soybean meal on growth performance, gut morphology, blood health, immune response, digestibility, digestive enzymes, and stress resistance. Aquacult Nutr. 2021;27(6):2612–28.

[66] Yossa R, Verdegem M. Misuse of multiple comparison tests and underuse of contrast procedures in aquaculture publications. Aquaculture. 2015;437:344–50.

[67] Moyo NAG, Rapatsa-Malatji MM. A review and meta-analysis of selected plant protein sources as a replacement of fishmeal in the diet of tilapias. Annals Anim Sci. 2023;23(3):681–90.

[68] Boyd CE, McNevin AA. 1 - Overview of aquaculture feeds: global impacts of ingredient production, manufacturing, and use. In: *Feed and Feeding Practices in Aquaculture (Second Edition).* edn. Edited by Davis DA. Oxford: Woodhead Publishing; 2022: 3–28.

[69] Mugwanya M, Dawood MAO, Kimera F, Sewilam H. Replacement of fish meal with fermented plant proteins in the aquafeed industry: a systematic review and meta-analysis. Reviews Aquaculture. 2023;15(1):62–88.

[70] Gule TT, Geremew A. Dietary strategies for Better Utilization of Aquafeeds in Tilapia Farming. Aquacult Nutr. 2022;2022(1):9463307.

[71] Midhun SJ, Arun D. Chap. 12 - Alternative feed technology in aquaculture. In: *Recent Advances in Aquaculture Microbial Technology.* edn. Edited by Mathew J, Jose MS, E. K R, Kumar A: Academic Press; 2023: 291–306.

[72] Borgeson TL, Racz VJ, Wilkie DC, White LJ, Drew MD. Effect of replacing fishmeal and oil with simple or complex mixtures of vegetable ingredients in diets fed to Nile tilapia (*Oreochromis niloticus*). Aquacult Nutr. 2006;12(2):141–9.

[73] Valente LMP, Cabral EM, Sousa V, Cunha LM, Fernandes JMO. Plant protein blends in diets for *Senegalese sole* affect skeletal muscle growth, flesh texture and the expression of related genes. Aquaculture. 2016;453:77–85.

[74] Chuang W-Y, Lin L-J, Shih H-D, Shy Y-M, Chang S-C, Lee T-T. The Potential Utilization of High-Fiber Agricultural By-Products as Monogastric Animal Feed and Feed Additives: A Review. In: *Animals.* vol. 11; 2021. 10.3390/ani11072098 PMC8300421 34359226

[75] Arome Ataguba G, Kamble MT, Salin KR. Food Industry By-Products as Protein Replacement in Aquaculture Diets of Tilapia and Catfish. In: *Food Processing By-Products and their Utilization.* edn.; 2017: 471–507.

[76] Duodu CP, Adjei-Boateng D, Amponsah AK, Andrews P, Obirikorang KA. The effect of plant protein-based diets on apparent nutrient digestibility, growth response, egesta quantity, postprandial ammonia excretion rate and serum quality of Nile tilapia. Aquac Res. 2020;51(3):1152–61.

[77] Awad A, Mohammady EY, Souady MR, Rabetimarghezar N, El-Haroun ER, Hassaan MS. Growth and physiological response of Nile tilapia (*Oreochromis niloticus*) fed a fermented mixture of plant protein sources. Anim Feed Sci Technol. 2024;315:116034.

[78] Mukhopadhyay R. Effect of fermentation on the nutritive value of sesame seed meal in the diets for rohu, *Labeo rohita* (Hamilton), fingerlings. Aquacult Nutr. 1999;5(4):229–36.

[79] Dawood MAO. Nutritional immunity of fish intestines: important insights for sustainable aquaculture. Reviews Aquaculture. 2021;13(1):642–63.

[80] Hekmatpour F, Nazemroaya S, Mousavi S-M, Amiri F, Feshalami MY, Sadr AS, Mortezavizadeh S-A, nejad LM, Houshmand H, Kianersi F, et al. Digestive function and serum biochemical parameters of juvenile *Cyprinus carpio* in response to substitution of dietary soybean meal with sesame seed (*Sesamum indicum*) cake. Aquaculture Rep. 2023;28:101438.

[81] Zhang H, Ran C, Teame T, Ding Q, Hoseinifar SH, Xie M, Zhang Z, Yang Y, Olsen RE, Gatlin DM, et al. Research progress on gut health of farmers teleost fish: a viewpoint concerning the intestinal mucosal barrier and the impact of its damage. Rev Fish Biol Fish. 2020;30(4):569–86.

[82] Abd El-Hack ME, El-Saadony MT, Nader MM, Salem HM, El-Tahan AM, Soliman SM, Khafaga AF. Effect of environmental factors on growth performance of Nile tilapia (Oreochromis niloticus). Int J Biometeorol. 2022;66(11):2183–94. 36044083 10.1007/s00484-022-02347-6 PMC9640449

[83] Ghaniem S, Nassef E, Zaineldin AI, Bakr A, Hegazi S. A comparison of the Beneficial effects of Inorganic, Organic, and Elemental Nano-selenium on Nile Tilapia: growth, immunity, oxidative status, gut morphology, and Immune Gene expression. Biol Trace Elem Res. 2022;200(12):5226–41. 35028868 10.1007/s12011-021-03075-5

[84] Zhang W, Xia S, Zhu J, Miao L, Ren M, Lin Y, Ge X, Sun S. Growth performance, physiological response and histology changes of juvenile blunt snout bream, Megalobrama amblycephala exposed to chronic ammonia. Aquaculture. 2019;506:424–36.

[85] Canosa LF, Bertucci JI. The effect of environmental stressors on growth in fish and its endocrine control. Front Endocrinol (Lausanne). 2023;14:1109461. 37065755 10.3389/fendo.2023.1109461 PMC10098185

[86] Iqbal M, Yaqub A, Ayub M. Effects of partial and full dietary substitution of fish meal and soybean meal by sunflower meal on growth performance, feed consumption, body indices, serum chemistry and intestine morphology of *Oreochromis niloticus*. Turkish J Fisheries Aquat Sci 2022, 22(10).

[87] Seibel H, Baßmann B, Rebl A. Blood will tell: what hematological analyses can reveal about Fish Welfare. Front Veterinary Sci 2021, 8. 10.3389/fvets.2021.616955 PMC8042153 33860003

[88] Dawood MAO, Noreldin AE, Sewilam H. Blood biochemical variables, antioxidative status, and histological features of intestinal, gill, and liver tissues of African catfish (*Clarias gariepinus*) exposed to high salinity and high-temperature stress. Environ Sci Pollut Res. 2022;29(37):56357–69. 10.1007/s11356-022-19702-0 PMC9374635 35338459

[89] Kalas MA, Chavez L, Leon M, Taweesedt PT, Surani S. Abnormal liver enzymes: a review for clinicians. World J Hepatol. 2021;13(11):1688–98. 34904038 10.4254/wjh.v13.i11.1688 PMC8637680

[90] Sheridan MA. Coordinate regulation of feeding, metabolism, and growth: perspectives from studies in fish. Gen Comp Endocrinol. 2021;312:113873. 34329604 10.1016/j.ygcen.2021.113873

[91] Triantaphyllopoulos KA, Cartas D, Miliou H. Factors influencing *GH* and *IGF-I* gene expression on growth in teleost fish: how can aquaculture industry benefit? Reviews Aquaculture. 2020;12(3):1637–62.

[92] Ayisi CL, Yamei C, Zhao J-L. Genes, transcription factors and enzymes involved in lipid metabolism in fin fish. Agri Gene. 2018;7:7–14.

[93] Tocher DR. Metabolism and functions of lipids and fatty acids in Teleost Fish. Rev Fish Sci. 2003;11(2):107–84.

[94] Kirrella AA, Abdo SE, El-Naggar K, Soliman MM, Aboelenin SM, Dawood MAO, Saleh AA. Use of Corn Silk Meal in broiler Diet: Effect on Growth Performance, Blood Biochemistry, immunological responses, and growth-related gene expression. In: Animals 11; 2021. 10.3390/ani11041170 PMC8073180 33921779

[95] Santos WM, Costa LS, López-Olmeda JF, Costa NCS, Santos FAC, Gamarano PG, Silva WS, Rosa PV, Luz RK, Ribeiro PAP. Effects of dietary protein levels on activities of protease and expression of ingestion and protein digestion-related genes in Nile tilapia juveniles. Aquac Res. 2020;51(7):2973–84.

[96] Francis G, Makkar HPS, Becker K. Antinutritional factors present in plant-derived alternate fish feed ingredients and their effects in fish. Aquaculture. 2001;199(3):197–227.

[97] Samtiya M, Aluko RE, Dhewa T. Plant food anti-nutritional factors and their reduction strategies: an overview. Food Prod Process Nutr. 2020;2(1):6.

[98] Hossain MS, Small BC, Kumar V, Hardy R. Utilization of functional feed additives to produce cost-effective, ecofriendly aquafeeds high in plant-based ingredients. Reviews Aquaculture. 2024;16(1):121–53.

[99] Siddik MAB, Julien BB, Islam SMM, Francis DS. Fermentation in aquafeed processing: achieving sustainability in feeds for global aquaculture production. Reviews Aquaculture. 2024;16(3):1244–65.

